# A Selective Nuclear Factor-κB Inhibitor, JSH-23, Exhibits Antidepressant-like Effects and Reduces Brain Inflammation in Rats

**DOI:** 10.3390/ph17101271

**Published:** 2024-09-26

**Authors:** Ahmad Nassar, Jacob Kaplanski, Abed N. Azab

**Affiliations:** 1 Department of Clinical Biochemistry and Pharmacology, Faculty of Health Sciences, Ben-Gurion University of the Negev, Beer-Sheva 8410501, Israel; 2 Department of Nursing, School for Community Health Professions, Faculty of Health Sciences, Ben-Gurion University of the Negev, Beer-Sheva 8410501, Israel

**Keywords:** brain, cytokines, depression, inflammation, mania, NF-κB

## Abstract

Background: Accumulating evidence suggests that nuclear factor (NF)-κB is involved in the pathophysiology of mood disorders. Objectives and Methods: We conducted two experimental protocols in rats to investigate the effects of a selective NF-κB inhibitor (JSH-23) on (i) lipopolysaccharide (LPS)-induced inflammation and (ii) on behavioral phenotypes in rat models of depression (sucrose consumption test and forced swim test) and mania (amphetamine-induced hyperactivity test). Additionally, we tested the effects of JSH-23 on levels of inflammatory components (interleukin-6, prostaglandin E2, nuclear phospho-p65, and tumor necrosis factor-α) in the brain. Results: Acute treatment with JSH-23 (10 mg/kg, intraperitoneally [ip]) led to potent anti-inflammatory effects in LPS-treated rats, including a diminished hypothermic response to LPS and a reduction in pro-inflammatory mediators’ levels in the brain. Chronic treatment with JSH-23 (3 mg/kg, ip, once daily, for 14 days) resulted in robust antidepressant-like effects (increased sucrose consumption and decreased immobility time). The antidepressant-like effects of JSH-23 were mostly accompanied by a reduction in levels of pro-inflammatory mediators in the brain. On the other hand, JSH-23 did not reduce amphetamine-induced hyperactivity. Conclusions: Altogether, these data suggest that NF-κB may be a potential therapeutic target for pharmacological interventions for depression.

## 1. Introduction

Psychiatric disorders impact millions of people worldwide, leading to unimaginable suffering and an enormous burden to patients and their families [1,2]. Among the most common and severe psychiatric illnesses are mood disorders such as depression and bipolar disorder; for instance, the prevalence of major depression and bipolar disorder is estimated at 11–15% [3,4] and 1–2% [5,6], respectively. Major depression is a devastating mental illness typically characterized by low mood and decreased functioning, among other features. Bipolar disorder is designated by recurrent bouts of mania, hypomania, and depression. Mood disorders negatively affect the functioning of affected subjects, impair the mental and social status of patients and their families, and are responsible for a huge burden and increased rate of comorbidities [1,2,7,8]. These figures underline the need for a continuous search for novel therapeutic strategies for patients with psychiatric disorders.

Ample evidence suggests that disruptions in immune system activity and alterations in inflammatory mediators’ production contribute to the pathophysiology of psychiatric disorders such as depression [9,10,11,12,13,14] and bipolar disorder [9,11,13,15,16], among others [14,17,18,19,20,21]. Several inflammatory components have been associated with the pathophysiology and treatment of mood disorders, including interleukin (IL)-6 [17,18,22], prostaglandin (PG) E2 [23,24,25,26], tumor necrosis factor (TNF)-α [17,22,25], and nuclear factor (NF)-κB [27,28,29]. Similar findings were repetitively reported by other investigators [17,18,22,27,28,30,31,32,33,34,35,36,37,38,39,40,41,42,43].

**Figure 1:**
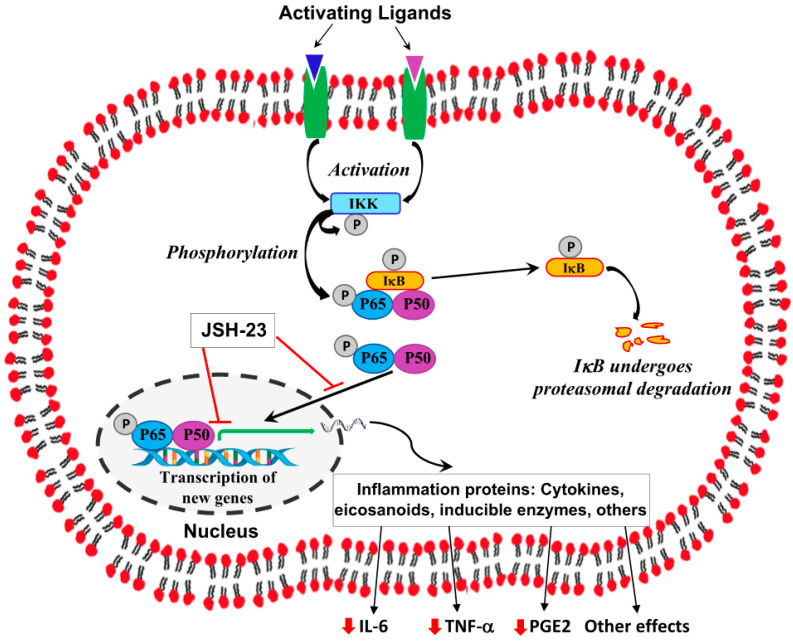
Depiction of the NF-κB machinery and site of action of JSH-23. An NF-κB heterodimer comprising p65 and p50 remains inactive in the cytoplasm while connected to I-κB. Following stimulation by different extracellular ligands, IKKs are activated and consecutively phosphorylate IκB and the p65-p50 dimer, leading to the separation of I-κB from the dimer. Then, the phosphorylated dimer transfers to the nucleus where it binds to distinct DNA sequences. The activation of these sequences regulates and activates the transcription of numerous genes related to the immune system, inflammation, cancer, and other cellular pathways. The selective inhibitor JSH-23 reduces the activity of NF-κB by blocking the entrance of phosphorylated dimers into the nucleus and by mitigating the transactivation of target genes. Black arrows indicate activation/phosphorylation/production, 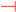 indicate inhibition/blocking; 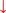 indicates reduction. Abbreviations: I-κB, inhibitor κB; IKK, I-κB kinase; IL, interleukin; P, phosphate; PG, prostaglandin; TNF, tumor necrosis factor.

NF-κB is a transcription factor that regulates multiple cellular pathways and is involved in the pathophysiological mechanisms underlying many diseases, particularly inflammation-associated conditions and cancer [44,45,46,47,48,49,50,51,52,53,54,55]. The NF-κB machinery exists in nearly all mammalian cells and is activated by various stimuli, such as infectious pathogens and their components (e.g., bacterial lipopolysaccharides [LPSs] and viral proteins), inflammatory mediators, as well as stress and ischemia-induced molecules. The NF-κB machinery comprises several proteins of the Rel family, including p50, p65 (RelA), and RelB [44,45,48,49,50,51]. At resting conditions, NF-κB proteins reside in the cytosol while they are bound to inhibitor-κB (I-κB), which inhibits their activity by masking the nuclear localization sequence and, thus, blocking their translocation to the nucleus [44,45,48,49,50,51]. Following activation by different activating ligands, IκB and NF-κB are phosphorylated by I-κB kinases (IKKs), leading to the dissociation of IκB from NF-κB. Then, IκB is poly-ubiquitinated and degraded in the proteasome, and NF-κB migrates to the nucleus [44,45,48,49,50,51]. Phosphorylation of NF-κB mostly occurs at multiple serine residues of the p65 subunit and plays a key role in nuclear translocation, DNA-binding activity, and the regulation of its transcriptional activity [56]. NF-κB proteins congregate to generate homo- or hetero-dimers that can enter the nucleus, bind to the DNA, and activate the transcription of target genes [44,45,48,49,50,51]. A simplified representation of the NF-κB pathway is presented in Figure 1.

In recent years, NF-κB has become a therapeutic target for multiple pharmacological interventions aimed at treating various illness states [57,58,59,60]. The benzenediamine derivate JSH-23 (4-methyl-N1-3-phenyl-propyl-benzene-1,2-diamine) is a selective NF-κB inhibitor; it directly inhibits the translocation of NF-κB to the nucleus, thereby diminishing its transcriptional activity [57,61,62] (see Figure 1 for illustration). On the other hand, JSH-23 does not seem to alter IκB degradation [61]. Treatment with JSH-23 was associated with potent anti-inflammatory activity [61,62,63,64,65]. As mentioned, NF-κB has been repetitively associated with the pathophysiology and therapy of affective ailments [28,34,35,37,38,66,67,68,69,70]. For example, Barbosa and colleagues [69] reported a notable rise in phosphorylated p65 protein levels in affectively stable bipolar patients compared to healthy subjects.

There are ample data suggesting that anti-inflammatory drugs exert positive behavioral effects in patients with mood disorders [31,71,72,73,74,75] and that psychotropic drugs confer strong anti-inflammatory properties [76,77,78,79,80]. Considering the potent anti-inflammatory effects of JSH-23 [61,62,64,65], it was reasonable to assume that it would also have positive mood-modulating effects. In line with this assumption, previous preclinical studies examined the antidepressant-like potential of JSH-23 revealing encouraging results [64,81]. For example, Wang et al. [64] demonstrated that JSH-23 exhibited significant antidepressant-like effects in “depressed” mice that were subjected to a chronic mild stress protocol. The antidepressant-like effects of JSH-23 were accompanied by a prominent reduction in inflammatory mediators’ levels in mice brains [64].

The primary objective of the present study was to examine the anti-manic-like potential of JSH-23. Hyperlocomotion/hyperactivity is a prominent feature that characterizes many bipolar patients during the manic phase. As far as we know, the anti-manic-like effects of JSH-23 have never been tested in animals. Thus, we used the amphetamine-induced hyperlocomotion test (AIHT) [82,83,84] to assess the anti-manic-like effect of JSH-23. Moreover, we tested the antidepressant-like ability of JSH-23 utilizing the depression-modeling paradigms—the sucrose consumption test (SCT) [59,84,85] and the forced swim test (FST) [83,84,85]. Furthermore, to reaffirm the anti-inflammatory activity of JSH-23, we tested its influence on brain concentrations of IL-6, PGE2, and TNF-α in LPS-treated rats (LPS-induced inflammation model).

## 2. Results

### 2.1. Effects of JSH-23 on LPS-Induced Inflammation

#### 2.1.1. Effect of JSH-23 on LPS-Induced Hypothermia

**Figure 2:**
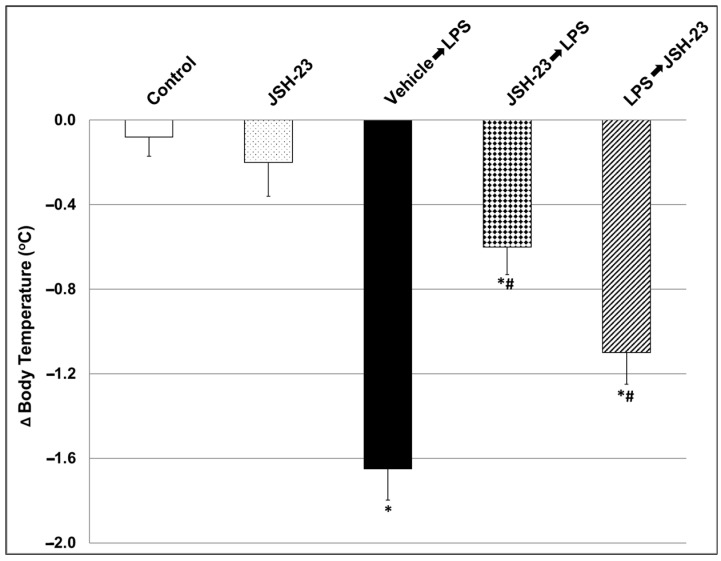
Effect of JSH-23 on LPS-induced hypothermia. Rats were treated intraperitoneally (ip) with vehicle or JSH-23 (10 mg/kg) at 2.5 h prior to LPS (1 mg/kg) injection. BT was assessed both prior to and at 1.5 h following the administration of LPS. The graph shows the change in BT after versus before (Δ) the injection of LPS. The values represent the average ± SEM for eight rats per group. One-way ANOVA test: F = 22.12, *p* < 0.0001; *t*-test: * *p* < 0.05 vs. Control group; # *p <* 0.05 vs. LPS group (Vehicle + LPS).

First, we tested the effects of JSH-23 (10 mg/kg) on LPS-induced hypothermia. As described in the Section 4, JSH-23 was administered at 2.5 h (h) before the endotoxin insult. As seen in Figure 2, the administration of LPS led to a prominent decrease in body temperature (BT) at 1.5 h post-injection (mean ± SEM: Control = −0.08 ± 0.09 vs. LPS = −1.6 ± 0.148, * *p* < 0.05). Pretreatment with JSH-23 significantly attenuated LPS-induced hypothermia (LPS = −1.6 ± 0.148 vs. JSH-23 + LPS = −0.6 ± 0.13, * *p* < 0.05).

#### 2.1.2. Effects of JSH-23 on Plasma Levels of IL-6, PGE2 and TNF-α in LPS-Treated Rats

**Figure 3:**
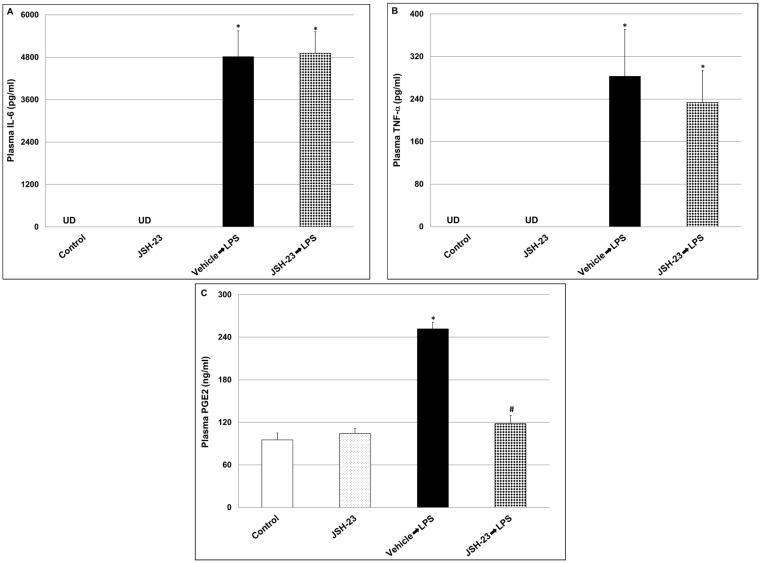
Effects of JSH-23 on plasma levels of IL-6, PGE2, and TNF-α in LPS-treated rats. Rats were treated with vehicle or JSH-23 (10 mg/kg) at 2.5 h before LPS (1 mg/kg) injection. At ~1.5 h after LPS injection, rats were euthanized, blood was collected, and plasma was separated. IL-6 (**A**), TNF-α (**B**), and PGE2 (**C**) levels were measured by ELISA. The values represent the average ± SEM for eight rats per group. (**A**) One-way ANOVA test: F = 35.11, *p* < 0.0001; *t*-test: * *p* < 0.05 vs. Control group. (**B**) One-way ANOVA test: F = 8.122, *p* = 0.0005; *t*-test: * *p* < 0.05 vs. Control group. (**C**) One-way ANOVA test: F = 59.49, *p* < 0.0001; *t*-test: * *p* < 0.05 vs. Control group; # *p <* 0.05 vs. LPS group. UD—undetectable.

The administration of LPS led to a significant increase in plasma levels of all three inflammatory mediators compared to the control group. Treatment with JSH-23 did not significantly alter plasma IL-6 (Figure 3A, LPS = 4816.1 ± 727.7 vs. JSH-23 + LPS = 4912 ± 607.6, *p* > 0.05) and TNF-α (Figure 3B, LPS = 283 ± 88 vs. JSH-23 + LPS = 233.8 ± 60, *p* > 0.05) levels. On the other hand, JSH-23 caused a significant decrease in plasma PGE2 levels (Figure 3C, LPS = 251.8 ± 9.4 vs. JSH-23 + LPS = 118.4 ± 11.3, *p* < 0.05).

#### 2.1.3. Effects of JSH-23 on Brain Levels of IL-6, PGE2, TNF-α and P-p65 in LPS-Treated Rats

**Figure 4:**
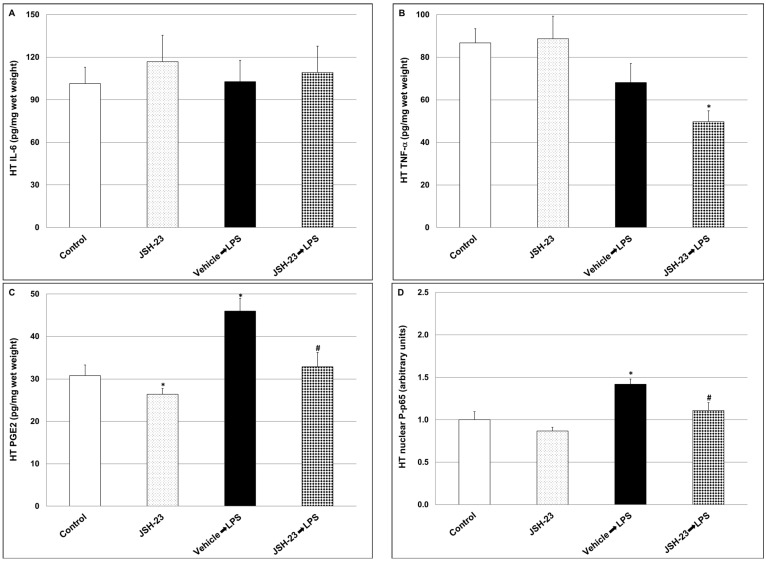
Effects of JSH-23 on hypothalamic levels of IL-6, PGE2, P-p65, and TNF-α in LPS-treated rats. Rats were treated with vehicle or JSH-23 (10 mg/kg) at 2.5 h before LPS (1 mg/kg) injection. At ~1.5 h after LPS injection, rats were euthanized, and their hypothalami extracted and processed as explained in the Section 4. Levels of IL-6 (**A**), TNF-α (**B**), PGE2 (**C**), and P-p65 (**D**) were determined using ELISA. The values represent the average ± SEM for eight rats per group. P-p65 levels are presented (arbitrary units) relative to their level in the control group, which is expressed as 1. (**A**) One-way ANOVA test: F = 0.1925, *p* = 0.9006. (**B**) One-way ANOVA test: F = 5.063, *p* = 0.0063; *t*-test: * *p* < 0.05 vs. Control group. (**C**) One-way ANOVA test: F = 10.79, *p* < 0.0001; *t*-test: * *p* < 0.05 vs. Control group; # *p <* 0.05 vs. LPS group. (**D**) One-way ANOVA test: F = 8.668, *p* = 0.0003; *t*-test: * *p* < 0.05 vs. Control group; # *p <* 0.05 vs. LPS group. HT—hypothalamus.

The levels of inflammatory mediators were determined in two brain regions—the hypothalamus (HT) and the hippocampus (HC). The administration of LPS did not significantly alter IL-6 and TNF-α levels in the HT (Figure 4A and Figure 4B, respectively; IL-6: Control = 101.4 ± 11.5 vs. LPS = 102.7 ± 15.1, *p* > 0.05, TNF-α: Control = 86.7 ± 6.7 vs. LPS = 68.1 ± 8.9, *p* > 0.05) but significantly increased PGE2 and nuclear phosphorylated-p65 (P-p65) (Figure 4C and Figure 4D, respectively; PGE2: Control = 30.8 ± 2.5 vs. LPS = 46 ± 3, *p* < 0.05, P-p65: Control = 1.00 ± 0.096 vs. LPS = 1.42 ± 0.06, *p* < 0.05). Treatment with JSH-23 reversed the LPS-induced increase in PGE2 and P-p65 levels (PGE2: LPS = 46 ± 3 vs. JSH-23 + LPS = 32.9 ± 3.3, *p* < 0.05, P-p65: LPS = 1.42 ± 0.06 vs. JSH-23 + LPS = 1.11 ± 0.092, *p* < 0.05). Moreover, JSH-23 significantly reduced TNF-α levels in LPS-treated rats (Figure 4B; LPS = 86.7 ± 6.7 vs. JSH-23 + LPS = 49.7 ± 5.1, *p* < 0.05), and PGE2 levels in vehicle-treated rats (Figure 4C; Control = 30.8 ± 2.5 vs. JSH-23 = 26.4 ± 1.4, *p* < 0.05).

**Figure 5:**
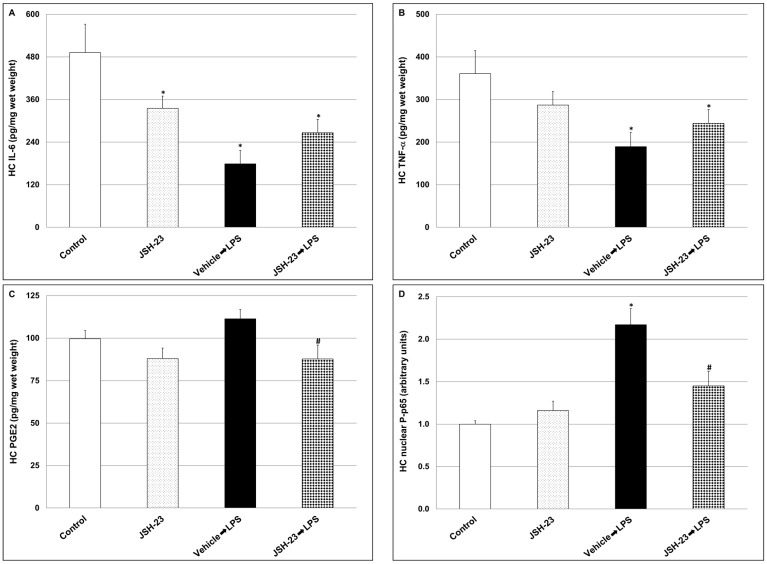
Effects of JSH-23 on hippocampal levels of IL-6, PGE2, P-p65, and TNF-α in LPS-treated rats. Rats were treated with vehicle or JSH-23 (10 mg/kg) at 2.5 h before LPS (1 mg/kg) injection. At ~1.5 h after LPS injection, rats were euthanized, and their hippocampi were extracted and processed as explained in the Section 4. Levels of IL-6 (**A**), TNF-α (**B**), PGE2 (**C**), and P-p65 (**D**) were determined using ELISA. The values represent the average ± SEM for eight rats per group. P-p65 levels are presented (arbitrary units) relative to their level in the control group, which is expressed as 1. (**A**) One-way ANOVA test: F = 6.789, *p* = 0.0014; *t*-test: * *p* < 0.05 vs. Control group. (**B**) One-way ANOVA test: F = 3.446, *p* = 0.03; *t*-test: * *p* < 0.05 vs. Control group. (**C**) One-way ANOVA test: F = 3.625, *p* < 0.025; *t*-test: # *p <* 0.05 vs. LPS group. (**D**) One-way ANOVA test: F = 17.24, *p* < 0.0001; *t*-test: * *p* < 0.05 vs. Control group; # *p <* 0.05 vs. LPS group. HC—hippocampus.

Furthermore, the administration of LPS led to a significant decrease in IL-6 and TNF-α levels in the HC (Figure 5A and Figure 5B, respectively; IL-6: Control = 492 ± 79.7 vs. LPS = 178.8 ± 37.3, *p* < 0.05, TNF-α: Control = 360.8 ± 53.8 vs. LPS = 189.1 ± 34, *p* < 0.05). On the other hand, LPS significantly increased P-p65 (Figure 5D; Control = 1.00 ± 0.04 vs. LPS = 2.17 ± 0.19, *p* < 0.05), and non-significantly increased PGE2 levels (Figure 5C; Control = 99.6 ± 4.8 vs. LPS = 111.4 ± 5.5, *p* > 0.05). Treatment with JSH-23 significantly reduced PGE2 and P-p65 levels in LPS-treated rats (Figure 5C and Figure 5D, respectively; PGE2: LPS = 111.4 ± 5.5 vs. JSH-23 + LPS = 87.7 ± 8.2, *p* < 0.05, P-p65: 2.17 ± 0.19 vs. JSH-23 + LPS = 1.45 ± 0.17, *p* < 0.05). Of note, JSH-23 significantly decreased HC IL-6 levels in control (non-LPS-treated) rats as well (Figure 5A; Control = 492 ± 79.7 vs. JSH-23 = 335 ± 34.7, *p* < 0.05).

### 2.2. Effects of Chronic JSH-23 Treatment on Behavioral Phenotypes and Brain Inflammation in Depression-like and Mania-like Models in Rats

Following the experiments that demonstrated that acute JSH-23 treatment confers protective effects against LPS-induced inflammation, we tested the effects of chronic JSH-23 treatment in rat models of depression and mania. Rats were treated with JSH-23 (3 mg/kg, ip), once daily, for 14–16 days, and behavioral tests were conducted (see Section 4). Moreover, these experiments aimed to determine the effects of chronic JSH-23 treatment on brain inflammatory mediators’ levels in rats subjected to non-pathogenic stress (forced swimming or amphetamine-induced hyperactivity) to assess whether JSH-23 possesses wide-range protective effects.

#### 2.2.1. Efficacy of Chronic JSH-23 Treatment in Depression-Modeling Paradigms

**Figure 6:**
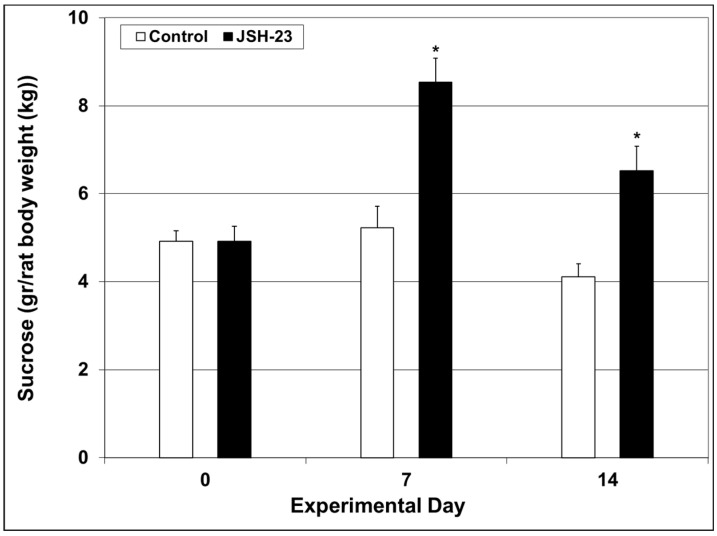
Effect of chronic JSH-23 treatment on sucrose consumption. Rats were treated with vehicle or JSH-23 (3 mg/kg) for 14 days. Sucrose consumption was calculated as follows: sucrose consumption (gr)/rats’ body weight (kg). The values represent the average ± SEM for 24 rats per group. *t*-test: * *p* < 0.05 vs. Control group, for the same time point.

Sucrose consumption test (SCT)—Before the initiation of JSH-23 treatment, rats were subjected to a baseline SCT (day 0). Thereafter, the SCT was repeated in the middle (day 7), and at the end of the treatment protocol (day 14). As seen in Figure 6, sucrose consumption was significantly higher in rats treated with JSH-23 after 7 (Control = 5.2 ± 0.49 vs. JSH-23 = 8.54 ± 0.55, *p* < 0.05) and 14 (Control = 4.11 ± 0.29 vs. JSH-23 = 6.52 ± 0.56, *p* < 0.05) days, suggestive of an antidepressant-like/hedonic-like effect of the NF-κB inhibitor.

**Figure 7:**
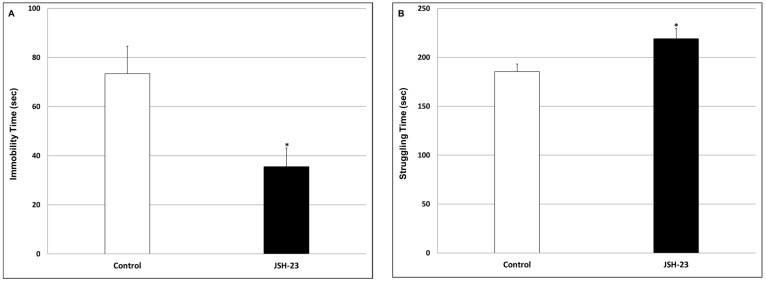
Effect of chronic JSH-23 treatment on immobility time and struggling behavior. Rats were treated with vehicle or JSH-23 (3 mg/kg) for 16 days and then subjected to an FST. The test lasted 5 min (min) and immobility time (**A**) and struggling (swimming and diving) time (**B**) were determined. The values represent the average ± SEM for 12 rats per group. *t*-test: * *p* < 0.05 vs. Control group.

Forced swim test (FST)—After 15 days of JSH-23 treatment, rats were subjected to an open field test (OFT) to assess their locomotor activity. This evaluation revealed that there were no significant differences between the groups. On the following day, at 4 h after JSH-23 injection, rats underwent an FST for assessing immobility time and struggling behavior. As seen in Figure 7, chronic pretreatment with JSH-23 significantly decreased immobility time (Figure 7A; Control = 73.4 ± 11.3 vs. JSH-23 = 35.6 ± 7.6, *p* < 0.05) and significantly increased struggling activity (Figure 7B; Control = 185.4 ± 7.7 vs. JSH-23 = 219.2 ± 10.5, *p* < 0.05), as compared to vehicle-treated rats. These findings attest to an antidepressant-like effect of JSH-23.

#### 2.2.2. Effects of Chronic JSH-23 Treatment on Brain Levels of IL-6, PGE2 and TNF-α in Post-FST Rats

**Figure 8:**
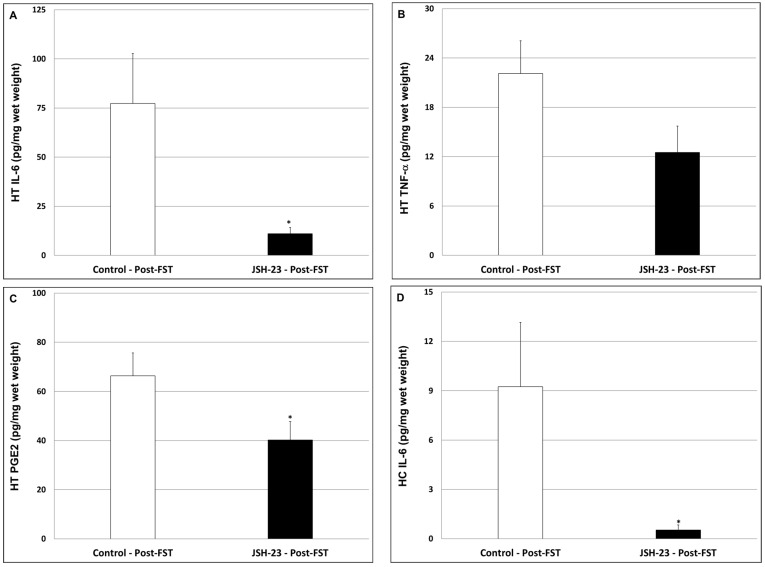

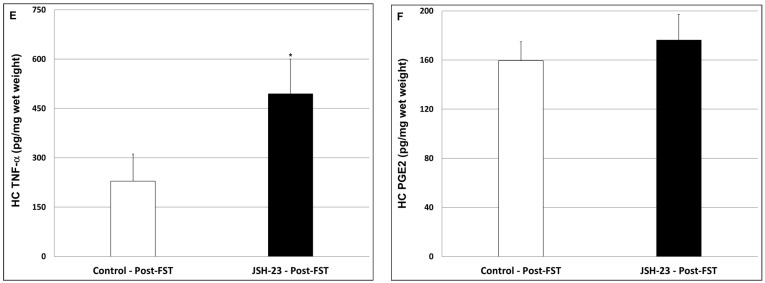
Effects of chronic JSH-23 treatment on brain levels of IL-6, PGE2, and TNF-α in post-FST rats. Rats were treated with vehicle or JSH-23 (3 mg/kg) for 16 days and then subjected to an FST. Thereafter, rats were euthanized and their hypothalami (**A**–**C**) and hippocampi (**D**–**F**) were extracted and processed as explained in the Section 4. Levels of IL-6 (**A**,**D**), TNF-α (**B**,**E**), and PGE2 (**C**,**F**) were determined using ELISA. The values represent the average ± SEM for 12 rats per group. *t*-test: * *p* < 0.05 vs. Control-post-FST group. HC—hippocampus; HT—hypothalamus.

As seen in Figure 8, JSH-23 significantly reduced HT IL-6 (A; Control = 77.3 ± 25.4 vs. JSH-23 = 11.1 ± 3.1, *p* < 0.05) and PGE2 (C; Control = 66.4 ± 9.3 vs. JSH-23 = 40.2 ± 7.5, *p* < 0.05) levels and led to a nearly significant decrease in TNF-α (Figure 8B; Control = 22.1 ± 4 vs. JSH-23 = 12.5 ± 3.2, *p* = 0.07). In the HC, JSH-23 significantly decreased IL-6 levels (Figure 8D; Control = 9.3 ± 3.9 vs. JSH-23 = 0.5 ± 0.3, *p* < 0.05) but had no effect on PGE2 (Figure 8F; Control = 159.4 ± 15.4 vs. JSH-23 = 176.2 ± 20.9, *p* > 0.05). Moreover, JSH-23 significantly increased HC TNF-α levels (Figure 8E; Control = 229 ± 82.3 vs. JSH-23 = 494.5 ± 105.4, *p* < 0.05). These results indicate that, overall, JSH-23 inhibited inflammatory mediators’ production in post-FST rats.

#### 2.2.3. Efficacy of Chronic JSH-23 Treatment in a Mania-Modeling Paradigm

**Figure 9:**
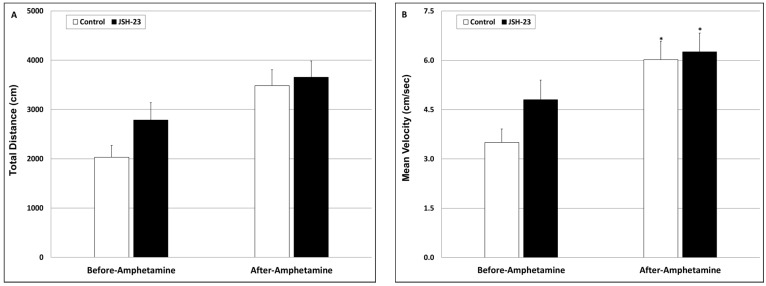
Effect of chronic JSH-23 treatment on amphetamine-induced hyperactivity. Rats were treated with vehicle or JSH-23 (3 mg/kg) for 15 days and then subjected to an initial OFT (which lasted 20 min) to assess their basic locomotor activity. On day 16, rats were injected with amphetamine (1 mg/kg, ip) and were again subjected to an OFT. Total distance (**A**) and mean velocity (**B**) during the last 10 min of the sessions were determined by a video-tracking system. The values represent the average ± SEM for 12 rats per group. (**A**) One-way ANOVA test: F = 1.458, *p* = 0.233; *t*-test: *p* = 0.063 vs. the same group before amphetamine injection. (**B**) One-way ANOVA test: F = 3.199, *p* = 0.0281; *t*-test: * *p* < 0.05 vs. the same group before amphetamine injection.

Amphetamine-induced hyperlocomotion test (AIHT)—After 15 days of JSH-23 treatment, rats were subjected to an OFT which revealed nonsignificant differences between the groups (Figure 9). On the next day, at 4 h after JSH-23 injection, rats were treated with amphetamine, which led to a nearly significant increase in total distance traveled (Figure 9A; Control pre-amphetamine = 2033.2 ± 238.2 vs. Control post-amphetamine = 3484.8 ± 320.2, *p* = 0.063) and a significant increase in mean velocity of movement (Figure 9B; Control pre-amphetamine = 3.5 ± 0.41 vs. Control post-amphetamine = 6.02 ± 0.6, *p* < 0.05). Pretreatment with JSH-23 did not significantly alter the distance traveled (Figure 9A; JSH-23 pre-amphetamine = 2787.7 ± 350.7 vs. JSH-23 post-amphetamine = 3656.7 ± 328.3, *p* > 0.05) and mean velocity (Figure 9B; JSH-23 pre-amphetamine = 4.8 ± 0.6 vs. JSH-23 post-amphetamine = 6.26 ± 0.57, *p* > 0.05) in amphetamine-treated rats. These findings suggest that JSH-23 lacks an anti-manic-like effect.

#### 2.2.4. Effects of Chronic JSH-23 Treatment on Brain Levels of IL-6, PGE2 and TNF-α in Post-AIHT Rats

**Figure 10:**
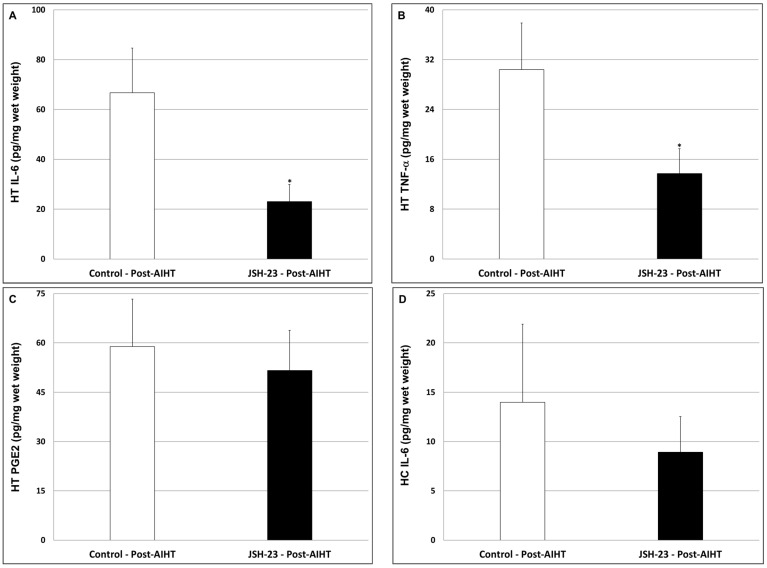

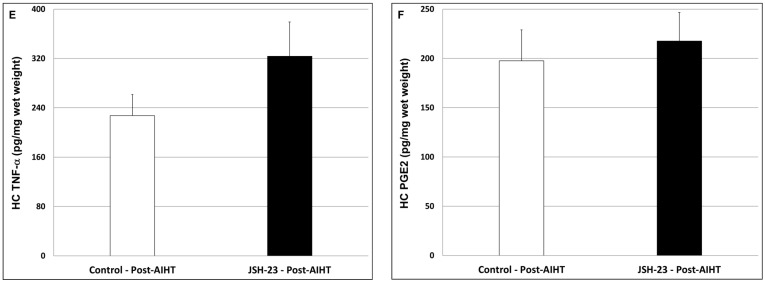
Effects of chronic JSH-23 treatment on brain levels of IL-6, PGE2, and TNF-α in post-AIHT rats. Rats were treated with vehicle or JSH-23 (3 mg/kg) for 16 days and then subjected to an AIHT. Thereafter, rats were euthanized, and their hypothalami (**A**–**C**) and hippocampi (**D**–**F**) were extracted and processed as explained in the Section 4. Levels of IL-6 (**A**,**D**), TNF-α (**B**,**E**), and PGE2 (**C**,**F**) were determined using ELISA. The values represent the average ± SEM for 12 rats per group. *t*-test: * *p* < 0.05 vs. Control-post-AIHT group. HC—hippocampus; HT—hypothalamus.

As seen in Figure 10, JSH-23 significantly decreased HT IL-6 (Figure 10A; Control = 66.7 ± 17.9 vs. JSH-23 = 23 ± 6.9, *p* < 0.05) and TNF-α (Figure 10B; Control = 30.4 ± 7.5 vs. JSH-23 = 13.7 ± 4, *p* < 0.05) levels but had no effect on PGE2 (Figure 10C; Control = 58.9 ± 14.4 vs. JSH-23 = 51.6 ± 12.2, *p* > 0.05). In the HC, JSH-23 did not significantly alter the levels of IL-6 (Figure 10D; Control = 14 ± 7.9 vs. JSH-23 = 8.9 ± 3.6, *p* > 0.05), TNF-α (Figure 10E; Control = 227.5 ± 34.1 vs. JSH-23 = 323.7 ± 55.5, *p* > 0.05) and PGE2 (Figure 10F; Control = 197.5 ± 31.5 vs. JSH-23 = 217.6 ± 28.9, *p* > 0.05).

## 3. Discussions

The major findings of the present study are as follows: (1) Acute treatment with JSH-23 (10 mg/kg) exhibited potent anti-inflammatory effects in LPS-treated rats. This included a diminished hypothermic response to LPS and a reduction in pro-inflammatory mediators’ levels in plasma and brain. (2) Chronic treatment with JSH-23 (3 mg/kg, once daily, for 14–16 days) exerted robust antidepressant-like effects (increased sucrose consumption in the SCT and decreased immobility time in the FST). The antidepressant-like effects of JSH-23 were accompanied, mostly, by a significant reduction in inflammatory mediators’ levels in the brain. (3) Chronic JSH-23 treatment did not reduce amphetamine-induced hyperactivity (as a model for mania-like behavior).

The immune-inflammatory response is a crucial, generally defensive process through which the mammalian body fights invading germs, removes abnormal cells and cell debris, and promotes tissue repair—all of which preserve homeostasis. Thus, inflammation is not necessarily a bad occurrence for the body, and its inhibition does not certainly benefit the involved organ/tissue. Nevertheless, an over-stimulated immune system and dysregulated inflammatory reactions, particularly if they last for a long time, may cause negative consequences, including disturbing symptoms, tissue damage, and organ dysfunction. Therefore, diverse anti-inflammatory approaches have been developed and used as possible therapeutic interferences against different types of inflammation. NF-κB plays a central role in various types of inflammatory processes [44,48,51,52,53,54].

Previous studies showed that systemic administration of LPS to mice and rats activates a prominent, complex immune-inflammatory response which stimulates multiple pathological processes including neuroinflammation and alterations in BT changes [82,86,87,88,89,90]. To specify, systemic administration of the endotoxin to rodents stimulates a short (1–2 h) hypothermia followed by a hyperthermia (fever) which may last between several hours to days [86,90]. In the present study, and consistent with earlier reports [86,90], we found that injection of LPS led to a significant decrease in rats’ BT, and that acute pretreatment with JSH-23 (10 mg/kg) significantly attenuated the hypothermic response to LPS (Figure 2). LPS-induced alterations in BT are usually associated with an increase in levels/activity of inflammatory components (such as IL-6, PGE2, TNF-α, and NF-κB) both in the plasma and in the brain, particularly in the HT [82,88,89,90,91,92]. As mentioned above, JSH-23 is a selective NF-κB inhibitor that attenuates the translocation of phospho-NF-κB-dimers to the nucleus and mitigates their transcriptional activity [57,61,62] (Figure 1). In the present study, the administration of LPS caused a significant elevation in plasma levels of IL-6, PGE2, and TNF-α (Figure 3). Pretreatment with JSH-23 mitigated the increase in plasma PGE2 levels but did not affect IL-6 and TNF-α in LPS-treated rats. Furthermore, LPS significantly increased PGE2 and nuclear P-p65 levels in the HT (Figure 4C,D) and P-p65 in the HC (Figure 5D)—these changes were significantly attenuated by pretreatment with JSH-23. The diminution of LPS-induced hypothermia by JSH-23 is probably associated with the observed decrease in HT PGE2 levels (Figure 4C) because the elevation in HT PGE2 levels is known to play a pivotal role in LPS-induced changes in BT [82,89,90,91]. Overall, these findings suggest that acute treatment with JSH-23 confers a protective effect against LPS-induced inflammation.

Regarding the mechanism underlying the hypothermic response to LPS, it is important to note that this response is not solely dependent on HT-PGE2 as other inflammatory mediators and cellular pathways are involved in this complex process [93,94]; For example, Steiner et al. [94] showed that the endocannabinoid cannabinoid-1 (CB1) receptor plays a role in the hypothermic response to LPS in rats. Systemic and intracerebroventricular (icv) administration of a CB1 antagonist—rimonabant, which originally was developed as an anti-obesity medication but was subsequently withdrawn from the market—significantly mitigated the LPS-induced hypothermia. Consistently, icv administration of the endocannabinoid anandamide—a CB1 agonist—significantly intensified the hypothermic response to LPS. Interestingly, the icv injection of rimonabant was associated with a significant decrease in plasma TNF-α levels in LPS-treated rats, suggesting that the anti-hypothermic effect of the CB1 antagonist may derive, at least in part, from the attenuation of TNF-α production [94].

Accumulating data suggest that NF-κB is involved in the pathophysiology of mood disorders [27,28,29,34,37,38,66,67,68,69,70] as well as other psychiatric illnesses [95,96,97,98,99]. Few previous studies examined the antidepressant-like potential of JSH-23 in behavioral models in animals revealing positive results [57,64,81]. The antidepressant-like effects of JSH-23 were accompanied by a prominent reduction in brain inflammatory mediators’ levels [64]. In the present study, we investigated the therapeutic potential of chronic JSH-23 treatment in depressive-like and mania-like models in rats. This study differs from previous studies in that it examined depressive-like behaviors (using the SCT and FST) in non-stressed rats. We assumed that it would be more difficult to demonstrate a beneficial effect in non-stressed animals because they will present less prominent depressive features. Nevertheless, the results clearly demonstrated that JSH-23 had significant antidepressant-like effects (Figure 6 and Figure 7). Additionally, JSH-23 caused a significant reduction in IL-6 levels in the HT and HC, and PGE2 levels in the HC in post-FST rats (Figure 8). These findings are in line with those of Wang et al. [64], who observed a reduction in hippocampal IL-6 in stress-subjected mice. However, we found that JSH-23 treatment significantly increased hippocampal TNF-α levels while they reported a significant decrease. This discrepancy may derive from the different designs of the studies (e.g., different treatment durations, and the use of non-stressed vs. stressed animals). It is worth noting that other studies also reported that NF-κB inhibition is associated with antidepressant-like properties [100,101,102]. For example, in mice subjected to chronic mild stress, simvastatin (a statin medication) exhibited antidepressant-like effects, which were attributed to its NF-κB inhibitory and anti-neuroinflammatory influence [102]. Similarly, Jiang et al. [100] reported that the plant product curcumin exhibited antidepressant-like effects in post-chronic mild stress rats, which were accompanied by potent anti-inflammatory/anti-NF-κB activity.

We are not aware of studies that investigated the efficacy of selective NF-κB inhibitors in mania-modeling paradigms. Therefore, we tested the impact of JSH-23 on amphetamine-induced hyperactivity in rats. We found that JSH-23 did not reduce the increase in locomotor activity in these animals (Figure 9), suggesting that it is not effective in this mania-like behavioral model. It is conceivable that a higher dose of JSH-23 (higher than the 3 mg/kg/day given in this study) would have resulted in a better anti-manic-like effect. We used a dose of 3 mg/kg/day based on previous studies that used chronic treatment protocols and demonstrated the beneficial behavioral effects of JSH-23 [64,81]. However, a higher dose does not necessarily improve the anti-manic-like effect of a given treatment compound [83]. Sun et al. [68] reported that the levels of NF-κB proteins were elevated in the postmortem frontal cortex of bipolar patients. In the present study, in post-AIHT rats, JSH-23 significantly decreased HT IL-6 and TNF-α levels but did not affect PGE2, whereas in the HC it lacked a significant influence on these inflammatory mediators (Figure 10). It is possible that the absence of a significant effect of JSH-23 on HT PGE2 levels contributes to its ineffectiveness against amphetamine-induced hyperactivity. In a previous study, we found that chronic treatment with *Nigella sativa* oil caused a significant reduction in amphetamine-induced hyperactivity (distance traveled and mean velocity) in male rats, which was accompanied by a significant decrease in HT PGE2 levels [84]. Interestingly, in that study [84], the *Nigella sativa* treatment did not impact the HT levels of IL-6 and TNF-α, raising the possibility that a reduction in HT PGE2 levels is more important for the anti-manic-like effect of the treatment. Consistent with this assumption, numerous preclinical and clinical studies reported that cyclooxygenase inhibitors—medications that inhibit the production of PGs, including HT PGE2 synthesis—conferred therapeutic benefits when they were administered alone or as an add-on therapy to manic bipolar patients [74,103,104,105] or animals subjected to mania-modeling tests [106,107,108].

The idea of selectively inhibiting a cellular pathway to treat a particular disease is tempting. However, in the case of NF-κB, one should bear in mind that this is a crucial cellular pathway essential for numerous physiological functions. Thus, potent inhibition of NF-κB may be harmful and lead to toxic effects [109,110,111,112]. It is worth noting that not all studies showed that NF-κB signaling is altered in psychiatric patients [113] and that under certain conditions activation of NF-κB may lead to therapeutic benefits [77,114]. Therefore, although NF-κB may be a potential therapeutic target for pharmacological interventions in psychiatric illness, more research is needed to elucidate the best pharmacological approach to alter the activity of this multi-functional cellular pathway. Particularly, it is important to determine which sub-groups of patients may benefit from NF-κB-modulating therapies and how to minimize the toxic influence of such interventions.

The present research has certain constraints. First, the study included only male rats. This is a significant limitation because extensive data show sex-associated differences in behavioral phenotypes across various rodent disease models [115,116,117], and in response to pharmacotherapy [118,119]. The inclusion of only male rats was based on the ethical principle of minimizing the number of animals needed for a “proof-of-concept” study while ensuring the ability to perform an adequate statistical analysis. Another limitation is the use of naïve rats (instead of those subjected to a stress protocol to induce a depression-like phenotype) for examining the antidepressive-like effects of JSH-23. However, it is important to accentuate that the major objective of this study was to explore the antimanic-like effect of JSH-23, not its antidepressant capability, which has been previously studied [57,64,81].

## 4. Materials and Methods

### 4.1. Animals

This study utilized wild-type male Sprague Dawley rats, each weighing between 220 and 250 g at the start of the experiments. Rats (purchased from Invigo R.M.S. Ltd.; Jerusalem, Israel) were grown, three per cage, under regulated conditions: ambient temperature 22 ± 1 °C, relative humidity 45–55%, photoperiod cycle 12 h light: 12 h dark, with free access to food and water ad libitum, unless stated otherwise. Only animals without evident pathology were included in the experiments. The procedures involving animal experiments were authorized by the Committee for the Use and Care of Laboratory Animals at Ben-Gurion University of the Negev, Israel (Authorization # IL-08-09-2014). At the start of the study, animals were assigned to the different experimental groups at random, and changes were performed solely to account for variations in the mean body weight among the groups.

### 4.2. Treatment with JSH-23

#### 4.2.1. Acute JSH-23 Treatment and Induction of Inflammation by LPS

**Figure 11:**
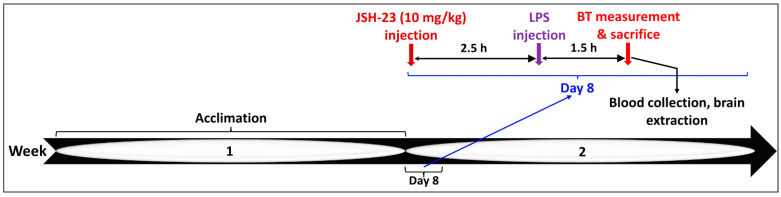
Timeline of the acute treatment with JSH-23 and induction of inflammation by LPS. Abbreviations: BT—body temperature, h—hour, LPS—lipopolysaccharide.

JSH-23 was purchased from Cayman Chemical (Ann Arbor, MI, USA). Previous *chronic* treatment protocols with JSH-23 used a daily dose of 3 mg/kg in rats and mice [63,64,81]. In this study, we conducted a preliminary experiment in which we examined the safety of two doses of JSH-23—3 and 10 mg/kg—given ip once daily, for a week, in rats. This experiment demonstrated that both doses were safe and did not negatively alter any of the basic physiological parameters that were tested (e.g., body weight, BT, social behavior. Therefore, in the *acute* treatment protocol, we decided to use a dose of 10 mg/kg JSH-23, given as a single injection after dissolution in dimethyl sulfoxide (DMSO, 0.1 mL). Control rats were treated with the vehicle. JSH-23 and DMSO were administered ip at 2.5 h before the injection of LPS (see Figure 11 for illustration). LPS from *Escherichia coli* (bought from Sigma-Aldrich; St. Louis, MI, USA) was dissolved in sterile NaCl 0.9% (0.1 mL/rat) and injected ip at a dose of 1 mg/kg to induce acute inflammation of a mild magnitude, similar to previous studies [82,86,87,88,89,90]. Control rats were injected with 0.1 mL NaCl 0.9%. BT was measured before and at 1.5 h after LPS injection (Figure 11). Rectal BT was determined using a highly sensitive thermometer (HL 600 Thermometer, Anritsu Meter Co., Tokyo, Japan); rats were acclimated to this procedure during the three days prior to the initiation of the experiment.

#### 4.2.2. Chronic JSH-23 Treatment in Rats Subjected to Behavioral Tests

**Figure 12:**
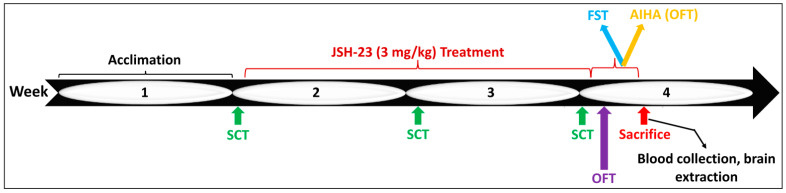
Timeline of the chronic JSH-23 treatment and the behavioral experiments. Abbreviations: AIHT—amphetamine-induced hyperlocomotion test, FST—forced swim test, OFT—open field test, SCT—sucrose consumption test.

We tested the effects of chronic treatment with JSH-23 in depression- and mania-like models in rats. Rats were injected ip once daily with either JSH-23 (3 mg/kg) [63,64,81] or DMSO (0.1 mL) for 14 days. At the beginning of these experiments, each treatment group (JSH-23 or vehicle) included 24 rats. To assess anhedonia/depressive-like behavior, rats were subjected to an SCT at baseline (day 0), in the middle (day 7), and at the end of the planned treatment regimen (day 14). On day 14, following the SCT, each treatment group was divided into two subgroups (*n* = 12 per group): one group was thereafter subjected to an FST, another was subjected to AIHT, and two corresponding control groups for each test (Figure 12). On day 13, an OFT was conducted to measure the basic locomotor activity of the animals, and on day 16, rats were subjected to either an FST or an AIHT (Figure 12). It is important to note that on days 15 and 16 of the treatment protocol, four hours before the conduction of the behavioral tests, rats received the daily treatment with JSH-23 or vehicle, to maintain the pharmacological effects of the interventions.

### 4.3. Behavioral Tests

All behavioral experiments were performed during the dark phase of the photoperiod cycle. Before the initiation of the behavioral studies, rats were acclimatized to housing conditions for one week and then subjected to the different behavioral tests (Figure 12).

#### 4.3.1. SCT

Usually, this behavioral paradigm is utilized to assess anhedonia/depressive-like behavior in animals that were pre-exposed to stress conditions to produce depressive-like features [83,85]. In the present study, we used naïve rats that were not subjected to stress conditions. Therefore, strictly speaking, here, depressive-like behavior was indirectly evaluated by measuring the hedonic-like comportment of the experimental animals [59,84]. The test was conducted, and sucrose consumption during a 24 h session was calculated as described previously [59,84,85]. The test was carried out under the same conditions at three time points as depicted in Figure 12.

#### 4.3.2. OFT

OFT evaluates the natural locomotion of the animals and is used as a controller for other behavioral assessments and may serve as an indicator of depressive-, anxious-, and manic-like behaviors [83,84,85,120,121]. Rats were placed for 20 min in an open-field arena (an unroofed black box: 60 cm [W] × 80 cm [L] × 60 cm [H]). A 5% ethanol solution was employed to sanitize the arena before introducing each animal. Sessions were recorded using a camera positioned about one meter overhead the center of the box, and, subsequently, the last 10 min of the sessions were examined using a video-tracking system (Ethovision, XT 14; Noldus Information Technology, Wageningen, The Netherland). The parameters that were analyzed are total distance traveled and scalar mean velocity of movement [83,84,85,118].

#### 4.3.3. Porsolt’s FST

The FST is a widely employed method for evaluating helplessness and depressive-like behavior in animals [83,84,85,120,122,123]. The test evaluates immobility time which signifies despair and passive-like behavior and struggling time, which represents active/fighting-like behavior. Immobility refers to the time during which the rat remains afloat in the water, doing only minimal movements necessary for retaining its head above the surface. Struggling behavior includes swimming and diving activities. On the test day (Figure 12), rats were subjected to a 5 min FST session which was videotaped. Subsequently, rats’ behaviors during the recorded sessions were evaluated by two independent experienced observers who were blind to the specific treatment of each rat [83].

#### 4.3.4. AIHT

This is a widely used paradigm for evaluating hyperactivity and mania-like behaviors in rodents [82,83,84,120,121,124]. On experiment day, rats were injected ip with amphetamine 1 mg/kg (D-amphetamine sulfate, Bulk 281, Bio-Techne Ltd., Abingdon, UK), and then placed in an open field arena for 20 min to measure their locomotor activity. Total distance traveled and mean velocity was analyzed for the last 10 min of the sessions as described above (Section 4.3.2).

### 4.4. Tissue Collection and Processing of the Samples

At the end of the experimental protocols (Figure 11 and Figure 12), rats were anesthetized using 4% isoflurane in pure oxygen and subsequently euthanized by decapitation. Thereafter, blood was collected into heparin-containing tubes for plasma separation, and brain regions (HT and HC) were taken out and processed as previously described [59,82,83,84,85,120]. The HT and HC were selected because they are associated with the pathological mechanisms underlying mood disorders [23,125,126,127]. The levels of IL-6, PGE2, P-p65, and TNF-α in the processed samples were quantified by specific ELISA kits (IL-6, PGE2, and TNF-α—kits from R&D Systems, Minneapolis, MN, USA; P-p65—a kit from eBioscience, San Diego, CA, USA) and analyzed according to previously described methods [83,84,85].

### 4.5. Statistical Analyses

Initially, the Shapiro–Wilk and D’Agostino-Pearson tests were used to determine if the examined variable was normally distributed. Accordingly, we used a one-way ANOVA followed by a two-tailed independent-samples *t*-test to assess between-group differences (for comparisons between ≥3 groups). Results are presented as average ± SEM based on the sample size specified in each figure. Statistical significance was defined as a *p*-value of less than 0.05. All figures depict the results of one out of two or three independent experiments which yielded similar outcomes.

## 5. Conclusions

This study shows that chronic treatment with the selective NF-κB inhibitor JSH-23 confers antidepressant-like effects, possibly due to the modulation of inflammatory processes in the brain.

## Data Availability

The results and data related to this publication are contained in the paper. The datasets used and/or analyzed in the study are available from the corresponding author upon reasonable request.
